# Anti-Dengue, Cytotoxicity, Antifungal, and* In Silico* Study of the Newly Synthesized 3-*O*-Phospo-α-*D*-Glucopyranuronic Acid Compound

**DOI:** 10.1155/2018/8648956

**Published:** 2018-12-03

**Authors:** Tareq Abu-Izneid, Abdur Rauf, Saud Bawazeer, Abdul Wadood, Seema Patel

**Affiliations:** ^1^Faculty of Pharmacy, Umm Al-Qura University, Makkah, Saudi Arabia; ^2^Department of Pharmaceutical Sciences, College of Pharmacy, Al Ain University of Science and Technology, Al Ain Campus, UAE; ^3^Department of Chemistry, University of Swabi, Khyber Pakhtunkhwa, Pakistan; ^4^Department of Pharmacy, Abdul Wali Khan University, Mardan 23200, Pakistan; ^5^Bioinformatics and Medical Informatics Research Center, San Diego State University, San Diego 92182, USA

## Abstract

The aim of the current study was to synthesize new bioactive compounds and evaluate their therapeutic relevance. The chemical structure of compound** 7** (methyl 3-O-phospo-*α*-D-glucopyranuronic acid was elucidated by physical and advance spectral technique. Also, this compound was assessed for various* in vitro* biological screening. The results showed that compound** 7** has promising antifungal activity against selected fungal strains. Computational study was also carried out to find antimalarial efficacy of the synthesized compounds. Compounds (**2-7**) were tested for cytotoxicity by MTT assay, and no considerable cytotoxicity was observed. Molecular docking study was performed to predict the binding modes of new compound (**7**). The docking results revealed that the compound has strong attraction towards the target protein, as characterized by good bonding networks. On the basis of the acquired results, it can be predicted that compound (**7**) might show good inhibitory activity against dengue envelope protein.

## 1. Introduction

Viruses are a major cause of diseases worldwide. Hepatitis C virus (HCV), human papillomavirus (HPV), influenza virus, herpes simplex virus (HSV), Japanese encephalitis virus, rotavirus, human immunodeficiency virus (HIV), respiratory syncytial virus (RSV), and Ebola virus are some of the most common pathogenic viruses. While HIV is largely contained in recent years, the emergence of dengue virus, Ebola virus, and Zika virus has been very alarming. Viruses bind to cell surface heparan sulfate proteoglycans and enter into the host cells. Viral proteases bind to host mannan binding lectins (MBL), the pattern recognition molecules, which leads to the activation of complement system [1–3]. The complement system includes serum proteins, receptors, and proteases. This system assists the antibodies and phagocytic cells in immune defense. But excess or perpetual activation of the complement system creates an inflammatory milieu and can cause blood coagulation.

Numerous phytochemicals have shown different degrees of inhibitory responses towards various pathogenic viruses. Polyphenols, alkaloids, sulfated polysaccharides, and glycosides among others have shown* in vitro* antiviral responses. In a study, cardiac glycosides inhibited HIV-1 gene expression, preventing the viral assembly, by manipulating Na-K-ATPase pump action [4]. Lanatoside C, a cardiac glycoside, has showed inhibitory effect on all four serotypes of dengue virus [5]. Polyphenolic compounds, flavonoids, chalcones, and other phenolics were the common docking ligands for dengue virus protein targets such as protease (NS2B-NS3pro), helicase (NS3 helicase), methyltransferase (MTase), RNA-dependent RNA polymerase (RdRp), and the dengue virus envelope protein [6]. Deacetyl-3-cinnamoyl-azadirachtin from* Azadirachta indica* inhibited NS3/4A protease of HCV [7].

Glucuronide or glucuronoside is glucuronic acid bound to other moieties by glycosidic bond. In fact, glucuronides are glycosides. Glucuronide derivatives constitute an important class of pharmaceutical active compounds which possess antiviral activity. Amantadine glucuronide and its derivative when combined with rimantadine inhibited the reproduction of influenza virus strains A/H1N1 and A/H3N2 [8]. Influenza virus inhibitory activities of spacer-linked 1-thioglucuronide analogues of glycyrrhizin have been observed [9].

In a study glucuronide 3-O-sulfated GlcA interfered with the binding of dengue virus to host cell receptors [10]. Further, this glucuronide had low cytotoxicity and it was effective at EC_50_ value lower than that of sucrose octasulfate, an inhibitor of dengue virus infection [10].

Hence, the aim of current finding was to synthesize methyl 3-O-phospo-*α*-D-glucopyranuronic acid, to conduct* in silico* analysis, and to predict pharmaceutical relevance.

## 2. Experimental

### 2.1. Material and Methods

The chemical and reagents used in this study were of analytical grade. The methylglucoside, 1-O-methyl glucose (**1**), was purchased from Sigma Aldrich. IR spectra were recorded on a FT-IR Nicolet 380 spectrophotometer (UK), as KBr disks, from 400-4000. ^1^H-NMR spectra were measured on AVANCE AV600 Crycroprob spectrometer in CDCl_3_. Mass spectra were recorded by using JEOL-600H-2 mass spectrometer; EI source 70 eV.

### 2.2. Synthesis of Methyl 3-O-Phospo-*α*-D-Glucopyranuronic Acid (7)

**Scheme 1:**
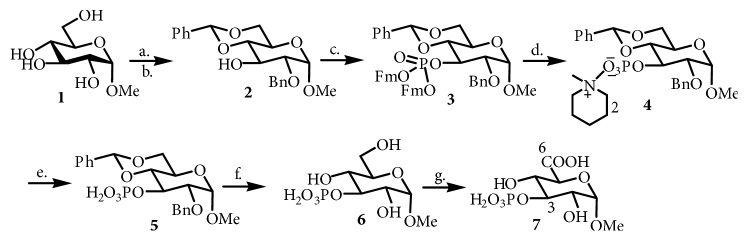
Synthesis of methyl 3-O-phospo-*α*-D-glucopyranuronic acid (**7**). Reagents: (a) ZnCl, rt (b) BnBr, NaH, THF, 0C (c)* i*Pr2NP-(OFm)2,* 1H*-tetrazole, H_2_O_2_,THF, (d) piperidine, CH_2_Cl_2_, (e) work-up with buffer, pH 6.0, (f) Et3SiH, 10% Pd/C, MeOH (g) NaOCl, NaOClO, piperidoxyl, acetonitrile, rt, pH 6.9.

Methyl 3-O-phospo-*α*-D-glucopyranuronic acid (**7**) (Scheme 1) was synthesized by using the following procedure. 1-O-Methyl glucose** 1** (1.00 mmol) was reacted with benzaldehyde (1.00 mmol) in tetrahydrofuran (THF; 1.00 mmol) as per standard procedure [10] and then reacted with benzyl chloride (1.00 mmol), in dimethylformamide (DMF; 1.00 mmol) in basic media (NaOH; 1.00 mmol) to obtain intermediate [11, 12]; then reaction mixture was refluxed for 48-72 h to obtain compound** 2**. To synthesize compound** 3**, compound** 2** (1.00 mmol) was reacted with di-Fm-phosphoramidite (1.00 mmol) in presence of 1*H*-tetrazole (1.00 mmol) and then oxidized with hydrogen peroxide [10]. Compound** 3** (1.00 mmol) is then reacted with pieridine to disconnect the Fm group, which presented compound** 4**. Compound** 4** after acidic work-up resulted in compound** 5**. Compound** 5 **(1.00 mmol) was reacted with triethylsilane and 10% Pd/C in methanol at 25°C followed by oxidation of primary alcohol [10], which generated methyl 3-O-phospo-*α*-D- glucopyranuronic acid (**7**) Yield; 700 mg (37%). The residue obtained in each step was purified by normal phase chromatography over silica gel by using chloroform: methanol as a solvent system. The purity of the compounds was confirmed by TLC. The structure of identified compounds was confirmed by comparing their spectral data with reported one [10].

### 2.3. Methyl 3-O-Phospo-*α*-D-Glucopyranuronic Acid (7)

White needle solid; yield: 90 mg, 65%, IR; 3443.50 for OH stretching, 2919.21 CH stretching, 2875.07 C=O stretching and 1682. 24, COOH stretching. EI-MS; 287, ^1^H-NMR (600 MHz, CDCl_3_): *δ*_H_ 5.01 (d, H-1,* J*=3.00), 3.58 (d, H-2.* J*=9.6), 4.36 (d, H-3,* J*=9.6), 3.56 (d, H-4,* J*=9.6), 4.56 (d, H-5,* J*=9.6) and 3.29 (s, OCH_3_).

## 3. Biological Activity

### 3.1. Cytotoxicity Study by MTT Methods

*In vitro* cytotoxicity study of the compounds (**2-7**) was performed by using mice hepatocytes and LCMK-2 monkey kidney epithelial cells [13]. Incubation of the compounds (**2-7**) was achieved in 24h and subsequently the cell ability was recognized by MTT (3-[4,5-dimethlthiazol-2yl]-2,5-diphenyltetrazolium bromide) method. In this procedure, the cells were kept in RPMI-1640 medium which was acquired from Gibco BRL. This medium comprises 110 *μ*g/ml penicillin sodium salt, 2 mg/ml sodium bicarbonate (Na_2_CO_3_), 10% fetal bovine serum (FBS), and 100 *μ*g/ml streptomycin sulfate. Initial seeding of the 7.1×10^3^ LCMK-2 cells and 8.6× 10^3^ mice hepatocytes was performed in 96-well plates. The cells were treated with the synthesized compounds (**2-7**) at different concentrations and also with a vehicle containing 0.2 % DMSO. Subsequently, they were subjected to incubation for 48h followed by conducting MTT assays.

### 3.2. Antifungal Activity

The antifungal properties of compounds (**2-7**) were assayed according to standard procedure. The tube dilution assay was performed to determine the fungal inhibitory effect of the compounds. Each compound was dissolved in DMSO at 2*μ*g/ml to prepare stock solution. SDA (Sabouraud dextrose agar) (4 ml) were poured into each tube and autoclaved for 15 min at 120°C and then allowed to cool at 15°C. To the SDA medium, stock solution (66.6 *μ*L) was added, which gave the final concentration 2mg/mL. All tubes were allowed to solidify in the slanted position at room temperature. Each tube was incubated with inoculum removed from a 7-day-old culture of fungal strain. The antifungal activity of each sample and standard drug was recorded after 7 days of incubation. The antifungal activity was performed in triplicate. Then the results were analyzed for the visible growth of fungi and % activity was calculated.

### 3.3. Molecular Docking

Molecular docking was carried out to predict the binding modes of newly synthesized 3-O-phosphated glucuronide derivative in the binding pockets of dengue virus envelope protein and antifungal enzymes. The 3D structures of dengue virus entry protein (PDB ID: 1OKE) and antifungal enzymes, dihydrofolate reductase (PDB ID: 4HOF), aspartic protease (PDB ID: 3Q70), and N-myristoyl transferase (PDB ID: 1IYL), were downloaded from protein databank (http://www.rcsb.org/) [14]. The 3D structures were then subjected to protonation and energy minimization, using default parameters of MOE (http://www.chemcomp.com/) [15]. The three-dimensional structure of 3-O-phosphated glucuronide derivative (**7**) was built by using Molecular Builder Module program implemented in MOE and saved as a (.mdb) file for molecular docking. Subsequently, the energy of the built compound was minimized up to 0.05 gradient using MMFF94s force field implemented in MOE. The modelled compound was docked into the active site of proteins using the Triangular Matching docking method (default) and 10 different conformations were generated. To obtain minimum energy structures, the ligand was allowed to be flexible during docking. At the end of docking, the predicted ligand-protein complexes were analyzed for molecular interactions.

## 4. Results and Discussion

### 4.1. Interaction of Compound with Dengue Envelope Protein

Previous research had showed that the crystal structure of soluble ectodomain of dengue virus 2 envelope has a hydrophobic pocket present in the hinge region between domains I and II [16]. This hydrophobic pocket binds to n-octyl-*β*-D-glucoside or *β*-OG (a small detergent molecule); therefore, it is known as *β*-OG binding site and it is proposed as an appropriate target for developing small molecule inhibitors of viral host fusion process [17]. This pocket is important for the low-pH-triggered conformational rearrangement during fusion [16]. So, compounds blocking the *β*-OG binding site interfere with conformational changes in the envelope protein [18]. The sequence similarity among four different dengue serotypes was carried out using NCBI BLAST. Result showed that the sequence of dengue 2 virus envelope protein 1OKE is almost identical with other DENV-2 sequences found in the NCBI website, with identity score about 99%. However, the identity of 1OKE sequence and other types of DENV sequences falls between 69% and 54%, with the least identity obtained when 1OKE was compared with the envelope protein of DENV-4. The higher the identity of two or more sequences, the more similar their protein structure is. If the target proteins share the identity of 50%, their protein structure is sufficiently reliable for drug design purpose [19].

**Figure 1:**
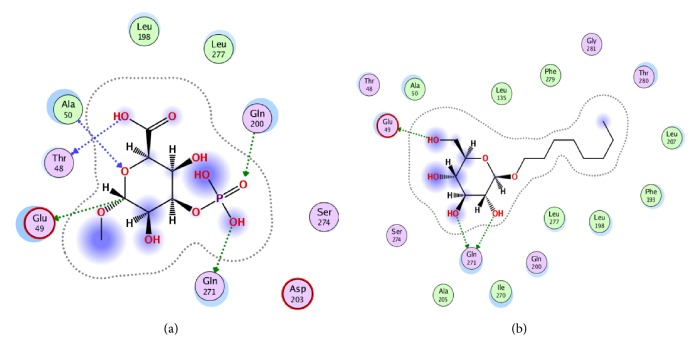
Binding mode of newly synthesized compound** 7** (a) and reference compound (**b**) in the active site of dengue envelope protein.

The docking results showed that the newly synthesized compound** 7** was well-accommodated in the binding pocket of *β*-OG. The docking scores for reference compound (octyl- *β*-OG) and compound** 7** were -7.0046 and -5.6691, respectively. The docking conformation of compound** 7 **in the hydrophobic pocket of envelope protein showed that this compound established five polar interactions and three hydrophobic interactions (Figure 1(a)). The polar interactions were established between the oxygen atoms of compound** 7 **and the active site residues Glu49, Thr48, Ala50, Gln200, and Gln271 of the envelope protein. Compound** 7** showed hydrophobic interactions with the nonpolar residues Ala50, Leu198, and Leu277 (Figure 1(a)). On comparison of the binding mode of the synthesized compound with the reference compound (cocrystallized compound), it was observed that both compounds showed about similar binding modes with dengue envelope protein (Figure 1(b)). The docking results showed that the synthesized compound has strong affinity with the target protein, as represented by good bonding networks with the target protein. On the basis of the obtained results, we can predict that this compound might show good inhibitory activity against dengue envelope protein.

### 4.2. Antifungal Activity

Antifungal activity of the synthesized compounds (**2-7**) was measured as per standard procedure. The results indicated that compounds** 2-7** exhibited excellent reduction in the growth of selected fungal strains. Among all tested compounds, compound** 7** exhibited maximum effect (35-45 mm) against* Aspergillus flavus, Candida albicans,* and* Fusarium solani. *Compounds** 2-6** showed moderate activity (10-35 mm) against selected fungal strains (Table 1). Hence, the results suggest environmentally friendly treatment avenue for fungal infections.

### 4.3. Docking against Antifungal Enzymes

To validate and specify the drug target for antifungal activity of these compounds, three different target proteins from* C. albicans* were selected as antifungal targets. Among them, only one target protein,* i.e*., dihydrofolate reductase (DHFR) from* C. albicans*, was preferred as it showed good docking score (-5.8605) and interactions with compound** 7**, as compared to secreted aspartic protease (4.9656) and N-myristoyl transferase (4.7592) in docking simulation.

**Figure 2:**
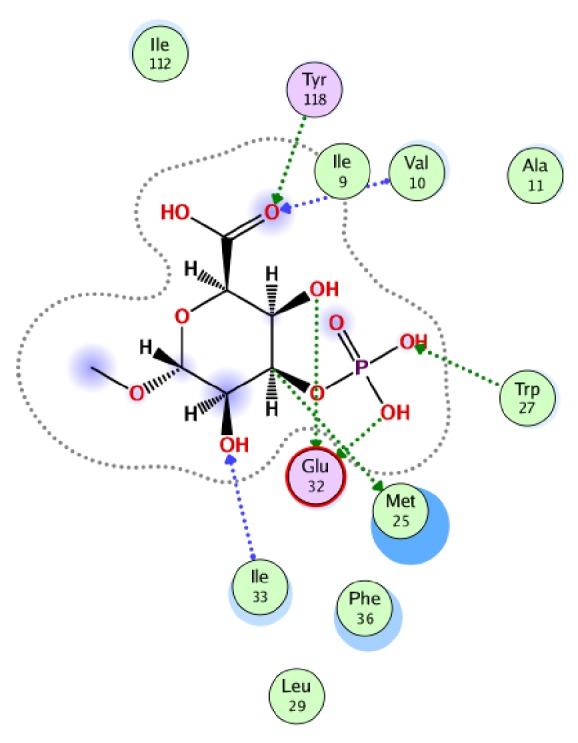
Binding mode of newly synthesized compound** 7** in the active site of DHFR.

The docking conformation of compound** 7** in the binding pocket of DHFR showed that this compound establishes seven hydrogen bonds with the active site residues Val10, Met25, Trp27, Glu32, Ile33, and Tyr118 of DHFR (Figure 2). This compound also formed hydrophobic interaction with active site residues Ala11, Leu29, Phe36, and Ile112. This strong bonding network might be one of the reasons for this compound's good antifungal activity.

### 4.4. Cytotoxic Effect

Compounds (**2-7**) were assessed for their cytotoxicity by using MTT assay. No considerable cytotoxicity was observed.

### 4.5. Antifungal Effect

The results of the antifungal activity of compounds (**2-7**) are illustrated in Table 1. Among all the tested compounds, compound** 7** showed good antifungal activity against* A. flavus, C. albicans*, and* F. solani *with % zone of inhibition which ranges in 35-45 mm, followed by compounds** 2-6 **(Table 1). The current finding demonstrated wide antifungal potency of compound** 7** followed by compounds** 2-6** against selected pathogenic fungi.

## 5. Conclusion

The current finding describes the synthesis, characterization,* in vitro* antifungal, cytotoxicity, and* in silico* anti-dengue study of methyl 3-O-phospo-*α*-D-glucopyranuronic acid** (7)**. The results showed that compound** 7** has promising antifungal activity against selected fungal strains. Cytotoxicity was negligible. The docking showed that compound (**7**) has strong attraction towards the target protein, characterized by good bonding networks. On the basis of the developed results, we can predict that compound (**7**) might show good inhibitory activity against dengue envelope protein.

## Figures and Tables

**Table 1:** Antifungal activity of compounds **2-7**.

**Name of the fungus**	%** Zone of inhibition (mm)**							
	**2**	**3**	**4**	**5**	**6**	**7**	STD	MIC (*μ*g/ml)
*A. flavus*	30 ± 1.44	30±1.14	25±1.48	28±1.88	35±2.06	44±1.16	Amphotericin B	20.20

*C. albicans*	-	-	-	-	-	45±1.17	Miconazole	110.8

*C. glabrata*	18±1.20	15±1.46	18±1.28	20±2.27	-	-	Miconazole	110.8

*F. solani*	-	-	20±1.20	15±1.88	10±2.00	35±1.49	Miconazole	110.2

*M. canis*	20±2.00	18±1.01	-	-	-	-	Miconazole	98.4

*T. logifusus*	15±1.24	30±2.02	-	-	-	-	Miconazole	100.5

## Data Availability

The data used to support the findings of this study are available from the corresponding author upon request.
